# Challenges and coping strategies when caring for terminally ill persons with cancer: perspectives of family caregivers

**DOI:** 10.1186/s12904-024-01518-z

**Published:** 2024-07-17

**Authors:** Antoinette Biney, Jacob Owusu Sarfo, Collins Atta Poku, David Atsu Deegbe, Fidelis Atibila, Gilbert Ti-enkawol Nachinab, Emmanuel Anaba, Gladys Dzansi, Priscilla Yeye Adumoah Attafuah

**Affiliations:** 1 Ghana College of Nurses and Midwives, Accra, Ghana; 2 Department of Health, Physical Education and Recreation, University of Cape Coast, Cape Coast, Ghana; 3 School of Nursing and Midwifery, Kwame Nkrumah University of Science and Technology, Kumasi, Ghana; 4 School of Nursing and Midwifery, University of Ghana, Legon, Accra Ghana; 5 School of Life and Medical sciences Centre for postgraduate medicine and Public Health, University of Hertfordshire UK, College Lane Campus, Hertfordshire, UK; 6 Department of General Nursing, School of Nursing and Midwifery, University for Development Studies, Tamale, Ghana; 7 School of Public Health, University of Ghana, Accra, Ghana

**Keywords:** Coping strategies, Family caregivers, Family management, Qualitative descriptive study, Supportive care, Terminally ill patients

## Abstract

**Introduction:**

Terminal illness is an irreversible illness that, without life-sustaining procedures, usually results in death or permanent disability from which recovery is unlikely. When involved, family caregivers are believed to improve health outcomes, such as reduced hospitalization, and establishing a patient’s initial access to professional treatment services. However, caring for a patient with a terminal illness is viewed as one of the most difficult aspects of providing care. This study aimed to identify the challenges, and coping strategies developed by family caregivers to cope with the care of the terminally ill person.

**Methods:**

An exploratory descriptive qualitative approach was used. Twenty (20) family caregivers voluntarily participated in the study from the Korle-Bu Teaching Hospital. Semi-structured interviews were conducted with the participants. The transcribed interviews were then analysed using thematic analysis.

**Results:**

From the analysis, three main themes emerged: challenges, coping strategies, and social support. These themes encompassed sixteen subthemes including financial burden, bad health conditions, faith and prayer, and support from health professionals. From the study, both male and female family caregivers narrated that providing care for sick relatives undergoing terminal disease is characterized as a daily duty demanding one’s time and fraught with emotional strain. In addition, even though it was a difficult job, family members who provided care for ailing relatives never gave up, citing responsibility, the importance of family, and religious beliefs as the primary motivations for doing so.

**Conclusion:**

The difficulties and demands of family caregiving roles for terminally ill relatives are complex and multifactorial. The findings call for multidisciplinary professional attention for family caregivers and policies that will support their lives holistically.

**Supplementary Information:**

The online version contains supplementary material available at 10.1186/s12904-024-01518-z.

## Introduction

The World Health Organization (WHO) [1] advises that patients with terminal illnesses receive palliative care to maximize their quality of life. According to the American Cancer Society [2], terminal illness is an irreversible condition that, without treatment, will certainly cause death soon or leave the patient in a permanently disabled state from which recovery is uncertain. One thing that stands out globally is the growing burden of a terminal illness and the possible strain it places on caregivers. In persons 70 years or older, the terminal illness burden is expected to rise by 183% between 2016 and 2060 [3]. Additionally, they predicted that by 2060, the current burden of terminal illness would have doubled, with low- and middle-income countries like Ghana experiencing the highest growth due to the adoption of certain lifestyles, especially dietary.

This provision of palliative care to terminally ill patients in high-income countries comparable to that of LMICs is wide. For example, Quinn et al. [4] revealed that most terminally ill patients in Canada receive palliative care compared to Pinto et al. [5] assertion that, only 17% of terminal illnesses adults in LMICs have access to palliative care. Several circumstances have been identified to hamper access to high-quality palliative care at LMICs. Among the factors is the key role of the family caregivers in a multidisciplinary team in ensuring that the terminally ill patient access palliative or end-of-life care [6, 7]. A multidisciplinary team approach is necessary for the care of terminally ill patients to maximize comfort by decreasing pain and attending to their physical, social, psychological, and spiritual needs [8, 9]. Members of the multidisciplinary team may include doctors, nurses, allied health professionals, volunteers, and family caregivers [10]. Especially in Ghana where the family plays a pivotal role in the care of ailing persons, family caregivers are very instrumental [11, 12]. Considering the inadequate staff on duty at a time, family members in most hospitals in Ghana, are directly involved in the personal care and sometimes feeding of patients [11–14].

Family caregivers are partners, relatives, or friends who devote significant time to providing healthcare over months or years and take on various physically, socially, emotionally, or financially taxing activities [15, 16]. Family caregivers provide care and support to patients who are terminally ill, yet doing so can harm their physical, psychological, and social well-being [7, 8, 17]. Effective family involvement in patient-centred care improves the standard of care provided [7, 8, 16–18].

Caregiving includes everything from helping the care recipient with everyday tasks and giving them direct care to navigating the complicated healthcare and social services systems. As an extension of the healthcare system and home-based care coordinators, family carers provide care [8, 19, 20] and perform all therapeutic and complex medical tasks, including ensuring effective adherence to treatment regimens [21]. Family involvement in health and medical tasks at home is not new, but it has become more common [22].

Several factors may influence family involvement in the care of the terminally ill. Stomski and Morrison [23] highlighted that involving family members could improve treatment decision-making by reducing communication barriers and emphasizing the needs of terminally ill patients and their caregivers. Caregivers usually establish a patient’s initial access to professional treatment services.

In Ghana, family caregivers have a particular set of challenges. While families in Ghana are becoming more nuclear and family support is decreasing [24], care for an ill family member still needs to be provided [24–26]. According to Shields [27], the degree or character of the family relationship influences family members’ willingness to participate as well as provide care [27]. In addition, financial issues, the caregiver’s health, connectedness, type of illness, and status of employment of the caregiver can all be considered [27, 28]. Despite the supportive role of family caregivers, various factors such as social expectations, the nature of family bonds, beliefs, and culture [29, 30] influence the involvement decision in various jurisdictions.

Numerous family members are usually delighted to assist [31] their ageing or ailing loved ones with the many health problems they encounter [28, 32]. However, these family caregivers are faced with several challenges that encompass their coping strategies, the dual system of rendering care as low-income countries are short-staffed, and their dual roles as caregivers in hospitals and at home [24–26, 33–35]. Essentially, the role of caring for family members may have both positive and negative ramifications that are far-reaching and ongoing for this category of caregivers [36]. Even though caregiving can be rewarding, it could cause significant disruptions to their lives and those closest to them, as caregiving demand can increase over time [37, 38].

Family involvement in the care of the terminally ill, especially the challenges they face and how they cope with these challenges in Ghana, is a grey area. The study aimed to identify the challenges family caregivers face and how they cope with the care of patients with terminal illnesses who are on admission or have been recently discharged at a teaching hospital in Ghana.

## Methods

### Design and setting

An exploratory descriptive qualitative approach was used to explore the in-depth experiences in-depth descriptions of the challenges and coping strategies of family caregivers for people with terminal illnesses. The goal of this design is to identify the characteristics of experiences that members of a specific demographic share. The study was carried out in the palliative care unit at the Korle-Bu Teaching Hospital (KBTH) family medicine department in Accra, Ghana. The KBTH serves as the main referral centre for southern Ghana and beyond.

### Population

The study focused on adult family caregivers who were the main caregivers for patients at the palliative care centre of the Family Medicine Department. Participants in this study were required to meet certain requirements to participate.

### Inclusion criteria

Any individual between the ages of 18 and 50 years, who takes care of his or her sick relative (whether in the nuclear or extended family; brother, mother, father sister, aunt, uncle, grandparents, or in-laws) who has a been diagnosed as having a terminal illness, that is one without cure, such as end-stage renal failure and advanced cancer (metastasized), and family caregivers caring for ill relatives for at least three months were included in the study.

#### Exclusion criteria

Participants who did not meet the selection criteria included family members of ill relatives receiving chemotherapy during the same period of the study, family members who did not live in the same house as the terminally ill relative, and family caregivers who were unwilling to participate.

### Data collection

To learn more about the participants’ challenges and coping mechanisms, a semi-structured interview guide with open-ended questions guided the conversation with each family caregiver. The first part of the guide gathered participant background data, including gender, age, educational attainment, employment status, and the duration of the care of the terminally ill family member. With the permission of each interviewee, audio recordings were made with an MP3 recorder, pen, and field notebook.

Family caregivers were identified at the Family Medicine Department of the KBTH when they came with their terminally ill relative to the unit for palliative care. Twenty-three (23) informal caregivers were identified as “family caregivers” and were given verbal and written information about the study and invited to participate. After reading through the consent form and explaining it verbally, twenty-two (22) family caregivers agreed and signed the consent document and indicated their interest in participating. After trying several times to book a date for the interview, 2 participants were never available. A total of 20 participants were interviewed. Participants included spouses, relatives, and relatives-in-law who were 18 years or older and provided care at home for their relatives with terminal illnesses such as advanced cancers or renal failure. The study was conducted following the principles of the Declaration of Helsinki for human research.

A semi-structured interview guide (see Appendix 1) was developed through literature and the adaptation of some questions from the palliative caregivers’ assessment tools [39] The interviews were conducted between April to July 2022. The conversations during the interview sessions were recorded with the consent of participants and later transcribed. Local languages (Akan and Ga languages) were used for participants who could not express themselves in English. The interviewer was fluent in both languages and transcribed such recordings with the last author to be sure the exact meanings were transcribed. To ensure that events were recorded consistently, an observation guide was implemented. The interviews took between 45 min and an hour per participant; the family caregivers were given some time, anywhere from 10 to 40 min, to deal with the reality of the situation when they were getting too emotional before resuming the conversation from where they had left off. Hence, some interviews lasted over an hour and a half whenever the participant became emotional. Data saturation was reached after the 20th participant, but 2 additional participants were being contacted to have their interviews done as they had already consented but were unavailable. As part of protection for participants’ vulnerability, psychological support care services were made available at the hospital.

### Data analysis

The analysis of the data in this study was conducted using the concepts of thematic analysis as outlined by Miles and Huberman [40]. This included data reduction, displaying the results, drawing conclusions, and verifying the results. Also, we addressed the issue of trustworthiness in the study to uphold the rigor of the qualitative research. Trustworthiness in qualitative research was established throughout the study by ensuring credibility, transferability, dependability, and confirmability [41].

For analysis, the recorded Akan and Ga interviews were transcribed with the help of a translator (without identifying the source of the data), and these transcriptions were examined with the participants to ensure that they were accurate representations of their descriptions. Alongside this process, field notes were also generated. After transcription, the transcripts were exported to the MAXQDA 2020 edition for analysis. One of the co-authors independently examined the transcripts. Then, two more authors received the findings and worked together to cross-check, discuss, and decide on themes and sub-themes. The authors were the only persons with access to the interview sessions. The data were stored in a computer protected by a password. Audio recordings will be destroyed five years after this study is completed and published.

## Results

Analysis showed that most family caregivers for patients with terminal illnesses were females (15). The male caregivers were either a spouse, a son, or a brother to a patient. Regarding the employment status of the caregivers, 15 of the participants were employed. The age range of family caregivers was between 28 and 58 years old. Regarding the participants’ religious backgrounds, 17 were Christians. The demographic characteristics of the participants are shown in Table 1, while Table 2 shows the characteristics of the patients they cared for. Data was analyzed, and the results were presented based on the themes and emerging sub-themes. Three (3) primary themes and sixteen sub-themes emerged from the subjective accounts of the experiences of the family caregivers. However, the focus of this article is the 3 main themes. The themes and their sub-themes, and the quotes are presented in Tables 3 and 4, and 5.

**Table 1:** Demographic characteristics of participants

CAREGIVER	GENDER	AGE	MARITAL STATUS	EMPLOYMENT STATUS	RELIGION	RELATION WITH PATIENT
Participant 1	F	56	Married	Self-employed	Christian	Daughter
Participant 2	F	37	Married	Trader	Christian	Sister
Participant 3	F	38	Married	Hairdresser	Christian	Wife
Participant 4	F	28	Married	Hairdresser	Christian	Daughter
Participant 5	F	42	Married	Trader	Christian	Daughter-in-law
Participant 6	F	54	Married	Teacher	Christian	Wife
Participant 7	F	45	Married	Trader	Muslim	Sister
Participant 8	M	58	Married	Prison Officer	Christian	Husband
Participant 9	F	50	Married	Insurance company	Christian	Wife
Participant 10	F	40	Married	Housewife	Christian	Mother
Participant 11	F	48	Married	Caterer	Christian	Daughter
Participant 12	M	35	Married	Media man	Christian	Son
Participant 13	F	22	Single	Student	Christian	Niece
Participant 14	M	54	Married	Un-employed	Christian	Brother
Participant 15	F	24	Single	Trader	Christian	Granddaughter
Participant 16	F	55	Divorced	Trader	Christian	Daughter
Participant 17	M	19	Single	Student	Muslim	Son
Participant 18	F	44	Widow	Banker	Christian	Sister
Participant 19	M	50	Married	Meat seller	Muslim	Son
Participant 20	F	58	Married	Housewife	Christian	Wife

**Table 2:** Distribution of patient characteristics

PARTICIPANT ID	DURATION OF INVOLVEMENT	PATIENT’S DIAGNOSIS	PATIENT’S AGE	GENDER	HEALTH STATUS (ECOG)
Participant 1	2years 4months	Breast Cancer	85	F	3
Participant 2	7 months	Breast Cancer	40	F	3
Participant 3	7years	Hepatocellular Carcinoma	40	F	3
Participant 4	9months	Hepatocellular Carcinoma	70	M	4
Participant 5	6months	Gastrointestinal Stroma Tumor (GIST)	90	F	4
Participant 6	2 years	Prostate Cancer	60	M	4
Participant 7	6months	Breast Cancer	40	F	4
Participant 8	3years 5mon	Ovarian Cancer	52	F	4
Participant 9	7months	Cholangio Carcinoma	53	M	2
Participant 10	1 year	Rhabdomyosarcoma	19	M	4
Participant 11	7months	Cervical Cancer	71	F	3
Participant 12	5months	Oesophageal Cancer	55	F	4
Participant 13	2 years	Breast cancer	59	F	3
Participant 14	8 months	Throat cancer	65	M	4
Participant 15	1 year	Lung cancer	70	F	4
Participant 16	3 years	Gastric cancer	80	F	4
Participant 17	6 months	Throat cancer	49	M	3
Participant 18	11 months	Breast cancer	38	F	3
Participant 19	7 months	Gastric Tumour	70	M	4
Participant 20	6 months	Hepatocellular carcinoma	60	M	3

**Table 3:** Sub-themes and some participants’ quotes from the theme Burden and challenges

THEMES	SUB-THEMES	QUOTES
Challenges	Financial Burden	*“My husband says he can’t pay for my mother’s treatment because he has other problems to solve*,* so I should ask my family for help. My sisters also don’t send any money because they say if I took over*,* it is because I think I can do it*,* so I should do it on my own.” ***48yrs**,** daughter** *“Things are very hard for me because with the provision shop*,* how much do I earn? How much at all do I get from it? And she* (the patient) *also has her provision shop. Even if we decide to put all the money together*,* it won’t be nearly as much for you to manage. I am not the type who borrows money*,* but that might be the only option I have now. To go for a loan”. ***56yrs**,** daughter** *“… I am drained. I sold my land to raise money for her treatment. As it stands now*,* any additional expenses may push me to go for a loan from my bank…”*. **44yrs**,** Sister** *“Financially*,* I think my resources or savings are almost depleted because her treatment has cost me a lot. I paid for all the medical procedures because my siblings are still in school; my father abandoned us when we were kids*,* so we don’t even know his existence. I don’t know what to do now”. ***35yrs**,** son**
	Fatigue and Stress	*“Sometimes I cry because of stress. …Not that he is sick*,* but I’m even stressing myself to take care of him. I am tired all the time and always feel light within myself.” ***38yrs**,** wife** *“… I will add that fatigue or stress are among my burden. Occasionally*,* I am unable to manage everything. I simply return to a state of calm when this occurs. Because if I’m tired*,* I always tell myself to rest so that when I have rested enough*,* I can do the work.” ***28yrs**,** daughter** *“Sometimes I am too tired to even wake up early and make sure she baths and takes her medicines on time. Stress is killing me. I am weak.” ***42yrs**,** daughter-in-law.** *“I don’t sleep*,* and he is always awake. He finds it difficult to sleep*,* so because of that*,* I also must stay awake … at night he will be calling you*,* “I am in pain. Give me something to stop the pain.” …”. ***54yrs**,** wife**
	Anxiety	*“Just two days ago*,* I got scared that she (mother-in-law) might die because she was in severe pain and kept talking about her two siblings who had already died. I became very anxious. Mostly*,* when I give her the morphine*,* she becomes relaxed and sleeps*,* and I was scared she might die while sleeping. For this reason*,* I didn’t give her the morphine. I waited for my husband and pretended to have forgotten so he would remind me. If anything happens to this woman with me alone in the house*,* I won’t know how to handle or explain it to them* (husband’s family).”**42yrs**,** daughter-in-law** *“…Sometimes*,* too*,* she can squeeze my hand and tell me*,* “Do not be scared*,* my son*,* death is there for everyone.” Whenever she makes such comments*,* I feel as though she is saying her goodbye.” ***35yrs**,** son**
	Bad health conditions	*“I have been taking medicine for hypertension*,* but since my husband’s condition changed or got worse; my BP has never dropped. I am always at 170/100 mm/Hg. I even forget to take my medicine sometimes.” ***54yrs**,** wife** *“I have been having severe knee discomfort*,* backaches*,* and hypertension I have not had it easy.*” **55yrs**,** daughter**
	Social isolation	*“… I’m not able to go to church. I can’t visit friends like I used to do. When I try to go to church and the church service is about to end*,* I begin to think of the problem at home. So*,* I prefer not to go anywhere.” ***28yrs**,** daughter** *“I am unable to allow my siblings to come to our house because my wife forbids me from involving any of my family members for fear that they will go and spread her condition. I don’t have any social life again. I’m always occupied with caring for her. I have stopped going to church for a while now.” ***58yrs**,** husband**
	Work challenges	*“Because of this*,* I have become slow in my business. I don’t take a lot of orders. At first*,* I was taking the orders and allowing the girls who work for me to take over*,* but they were spoiling it for me*,* so now I take only small orders*,* which is not very helpful because now I’m losing a lot of money.” ***48yrs**,** daughter** *“I used to gain some small funds from playing the keyboard at church and other functions*,* but currently*,* I don’t have such opportunities anymore.” ***35yrs**,** son** *“…*,* as I mentioned earlier*,* I had to close my shop*,* stop selling the “kente” and stay home to take care of my mother-in-law. Salesgirls are not trustworthy lately”. ***45yrs**,** daughter-in-law** *“I am a prison officer*,* but because of her disease*,* I took one year of leave without pay to take care of her. The work is such that one must be at the post every day*,* and I couldn’t do it when the disease started”. ***58yrs**,** husband**
	Family conflicts	*“…*,* it is not easy caring for him. He doesn’t understand why I should tell him what to do when he is old enough. I don’t sleep; he usually sleeps a lot during the day when I am at work. He can stay awake all night because of pain*,* and when I suggest to him to bear with me a bit so I can also catch some sleep before the next morning*,* which is a working day for me*,* he gets offended. He can shout at me and tell me to my face that I am being insensitive. Can you imagine?” ***50yrs**,** wife** *“I don’t have time for the kids*,* but it is not much of an issue. However*,* my husband is my burden. Sometimes he says I am using his mother as an excuse not to satisfy him sexually.” ***42yrs**,** daughter-in-law**
	Knowledge of relatives about the condition	*“I don’t like the fact that the doctors are not telling me what is happening to my son. They haven’t explained to me what exactly the problem is; they just said it is a sarcoma. What is sarcoma? I don’t even know what that means. Nothing was said about how it happened or why they couldn’t perform the surgery. …They also keep prescribing medicine for my son*,* but his condition is not improving. He is still in pain and the swelling is getting bigger and bigger and I cannot do anything about it. That is my worry now.” ***40yrs**,** mother** *“When she was first told she had cancer*,* we were not taught what type of food to eat and what not to eat*,* so I was finding it difficult to cook the right food for her. I would cook two separate meals*,* not even knowing which one was okay for her to eat. Till now*,* the doctors and nurses haven’t said anything. I asked some people to help me with the diet issues*,* so I am on it.” ***42yrs**,** daughter-in-law**

**Table 4:** Sub-themes and some participants’ quotes from the theme coping strategies

Coping Strategies	Faith and prayer	“…, but when I told my pastor’s wife about it, she motivated me to go ahead and not listen to the insults. She said we all have a cross to carry. Maybe mine is like this. I should focus and keep on praying and she will remember me in prayers and inform the prayer group in the church about it”.48yrs, daughter “I just have faith in God”.28yrs, daughter “To get rid of all the plenty thoughts in my mind, … I also read the Quran and recite my tasbih. …Whenever I start crying then I pick up my Tasbih and start reciting”.45yrs, sister
	Adjusting to the situations	*“…*,* There is no cure*,* but I am managing and adjusting to the situation. Each day that comes and passes is a blessing. Indeed*,* I am managing*,* and I don’t speak to the doctors anymore because it’s the same thing they will tell you every time…” ***28yrs**,** daughter** *“(*It’s a problem*) Coping! … Well*,* I can deal with some of them*,* but in others*,* I’m just like a piece of paper*,* going wherever the wind takes me. I am powerless to resist. I can therefore say that I have a choice given the stress issues at hand. When I’m exhausted*,* I’ll lie down and sleep. I can choose that option. …I can occasionally decide to disregard work when I am drained instead of doing it*,* I will go to bed. I balance my options and prioritize which tasks I need to complete for my father*,* my family*,* and myself”. ***28yrs**,** daughter** *“I try not to keep the environment too quiet; otherwise*,* he starts to get angry. I adopted another sleep pattern*,* even though it is not enough. When the health aid and my son are around*,* we perform duties in turns. …*,* we share the time we each stay with him unless he makes some gestures asking for me”. ***54yrs**,** wife** *“I also chat with her and try to recollect some of our childhood memories*,* the doctor said that can help my sister and it helps me as well. So normally chatting with her this way helps me a lot and I forget that she is even sick at times. When I am happy*,* I like to tell stories.” ***45yrs**,** sister**
	Leisure	*“I listen to some gospel tunes to encourage myself. My home speakers are always on because my husband loves music and so do I. I try not to keep the environment too quiet; otherwise*,* he starts getting angry. So*,* for music*,* it has helped a lot in this situation. … at times*,* depending on the weather and the level of pain*,* he demands a particular genre of music. But mostly*,* we listen to gospel and reggae.” ***50yrs**,** wife** *“I do a lot of video calls with my wife and children when they are not around*,* so I keep in contact with them. My wife also calls me on a video call to encourage me that there are blessings in what I am doing. I call in to see my kids and ask if they need anything and tell them that they will see me soon and that everything will be over soon. Grandma will be fine.* (Bows his head and shakes it).*” ***35yrs**,** son** *“I lie on the bed and take my mind off all these issues before I can sleep. I must relax. I need it. When I am awake*,* I feel so good*,* and I regain most of my lost energy. This is how I cope with the ones I have control over”. ***56yrs**,** daughter**
	Support system	*“My pastor’s wife is always calling to encourage me and pray with me as well*,* and I’m grateful for that. Even though it’s hard to know your mother is dying*,* I think it’s better than not knowing. My pastor’s wife told some of the prayer warriors in our church to check up on me and to call me to pray with me always”. ***48yrs**,** daughter**

**Table 5:** Sub-themes and some participants’ quotes from the theme of social support

Social Support	Support from health professionals	“The nurses help me a lot. They ask me sometimes if I need any help. I should feel free to inform them. All this depends on the good ones on duty (she burst into laughter slightly).” 28yrs, daughter “The doctors told me what the condition was and what to expect. On our last appointment, which was two weeks ago, we met a psychologist, who spoke to us at length preparing us for whatever might happen.”42yrs, daughter-in-law “The nurses and doctors at the palliative unit have time for us when we come for review; they also call us from time to time to check up on us. I ask a lot of questions and they take their time explaining everything to me.” 58yrs, husband “The hospital staff are also helpful because when we come for review, they always explain to us what to expect. Even though it’s hard to know your mother is dying, I think it’s better than not knowing. Since they know our condition when we get here, they make everything fast for us”.48yrs, daughter
	Support from colleagues and peers	*“I sell cosmetics and my colleague does cosmetology. I sometimes leave my shop in her care since most of her clients buy from me*,* and so I can make some money while I’m away and she can also retain all her customers.” ***37yrs**,** sister**
	Support from families	*“My cousin*,* who is a doctor*,* helps me a lot. She takes her on hospital rounds sometimes. My cousin*,* my uncle*,* and my husband have helped a lot*,* especially my cousin. When we come to the hospital and there is a drug to buy*,* she just picks it up and buys the drug. Sometimes I don’t ask for money*,* but they just give it like that. The one that surprises me is even my sick mother. … I don’t want to talk much*”.**56yrs**,** daughter** “*My children and husband support me by attending to my father when I may be doing something. Sometimes I instruct my children to do other things*,* and they are always available. I have observed for some time now*,* they (*children) *stay at home and even ask Mummy*,* “Can I help you with something?“ ***37yrs**,** sister**
	Support from religious groups and Representatives	*“Like I said earlier*,* our doctor at the hospital is also our pastor at church*,* he has been supporting us… he helps in prayers*,* he has informed the church about it and requested they pray for me and my husband all the time. The financial support is coming from the pastor and the church as well”. ***38yrs**,** wife** “*Every Friday evening*,* our Imam visits us at home to offer prayers for me and my sister. Sometimes he recites the Quran*,* and other times when he is unable to come*,* he sends a representative to come to us on his behalf. His representative will always encourage me that Allah is in this*,* so I shouldn’t worry (*she burst into tears and smiled a little after*) At times*,* some of the believers come to see us when we are home*,* especially on Fridays*,* to encourage us.” ***45yrs**,** sister** *“… the chief Imam in our community organizes regular prayer sessions for my father in our house whenever he is called upon.” ***50yrs**,** son**

## Discussion

This study aimed to identify the challenges and coping strategies developed by family caregivers to cope with the care of the terminally ill person. Out of the themes that emerged, we observed patterns of family caregivers’ challenges, coping strategies, and social support. As part of family caregivers’ challenges, seeing a loved one at the end of their life can be stressful and daunting. In the Ghanaian setup, most family caregivers are immediate relatives of the terminally ill patient. The analysis of the data shows that family caregivers are under pressure to take care of their sick loved ones and deal with a variety of responsibilities and challenges. This included debt, exhaustion, anxiety, and occasionally even health problems. Additionally, research indicates that carers experience family conflicts, social isolation, and a lack of understanding of the management pathway [6, 7, 9, 42, 43]. Most participants claimed they were happy to care for their relative despite all the challenges stated, but they did acknowledge there were occasions when they wanted to quit. These findings align with the findings of studies [44, 45] conducted in Ghana among family caregivers of people living with chronic conditions like mental illness.

In the current study, most family caregivers were female spouses, daughters, or sons of the care recipient. This is consistent with the conclusions of studies done by Ocansey et al., [45] and Kyei-Arthur and Codjoe [46], which showed that women are more likely than men to care for terminally sick family members. Furthermore, cultural norms that consider providing care to be a feminine responsibility [46–48] are more likely to be in Africa [49, 50]. Countries and cultures like Ghana uphold the traditional role of the woman as the primary caregiver [48]. Despite this, in recent times, more males are assuming caregiving roles, particularly husbands and sometimes sons [51–53]. This is reflected in the current study, in which four males took on the caregiving roles as the care recipient’s sons, brother, and husband. Thus, the male involvement in the care of terminally ill relatives shows a changing social norm in Ghana regarding the perception that the caregiver role is primarily reserved for females.

Taking care of family members with terminal illnesses, as depicted by the participants in the study, is not an easy task. The responsibilities they had assumed significantly impacted each participant’s personal life. This is supported by studies by Irfan et al. [54], Pope et al. [55], Ajibade et al. [56], and Bouldin et al. [57] that looked at family caregivers and found that they experienced personal life effects, financial difficulties, and decreased work productivity because of their role as caregivers. Participants in this study said they did not receive enough sleep to replenish the energy they had lost throughout the day. This resulted in ongoing weariness. Participants acknowledged that a main source of stress for them is their health decline. The stress and exhaustion they experienced because of their duties as caretakers had a detrimental effect on their health. They indicated they had started having knee discomfort, backaches, and hypertension. Long-term exposure to stress brought on by the duties of caregiving [58, 59] may have an impact on the daily operations of the family as well as the health of its members, according to research done by Ocansey et al. [45]. Similarly, it has been demonstrated in recent studies by Roth et al. [60] and Irfan et al. [54] that physically demanding caregiving roles might have a detrimental effect on caregivers’ health.

The participants’ social lives were disturbed because of the type of caregiving role they performed, which made it impossible for them to move around or participate in scheduled activities and events. The health of their ailing relatives led participants to admit that they frequently forbade visitors from visiting their homes. Their participation in social activities was impacted by this. Even if it were possible, they wouldn’t be able to take pleasure in the social activity since they wouldn’t feel at ease. This was in line with a study conducted by Lu et al. [50] in which family carers regarded their caregiving duties as “living on the edge and being prisoners in their own lives”. This finding is consistent with a previous study by Cleary et al. [61], which found that carers faced a great deal of instability, a rapid loss of health, and feelings of isolation.

The caregiving responsibilities can also impact family connections and life quality [62, 63]. The carers’ roles greatly impacted the connections between the family members. Maintaining open lines of communication while having time for their partners and children proved to be a huge challenge for them. As a result, family caregivers sacrificed their time, finances, and relationships with their children, spouses, and social aspects of their lives as they provided care. Participants complained that their caring tasks were so challenging that they were too worn out to carry on with their daily activities and care for their families. This supported a study by Pope et al. [55] that found that most family carers struggle to balance their multiple tasks, especially young adult caregivers who face the rare burden of caring for a family member while also meeting developmental milestones.

This challenge was compounded by the lack of knowledge on the condition of ill relatives. Participants lamented that health professionals hardly told them about the treatment modalities and progress of their ill relatives. They felt they were always kept in the dark and could not ask questions for fear of the outcome either to them or their relative. This supports a study by Tarberg et al. [18] on the “silent voices of caregivers”. In their study, Ullrich et al. [63] also noted that the knowledge demands of family carers were the highest. Their participants sought truthful answers to their questions, updates on changes in the patient’s condition, and information on exactly what was being done to the patient.

Aside the challenges, participants devised ways to cope with their current situations. One way to describe coping is as a person’s reaction to upsetting situations. From this study, family caregivers made use of music, video chats, relaxation, situational adaptability, and faith and prayer. One important feature of the study, video calls, was unique from those found in past studies. According to Cook et al. [64], Kim, & Dvorak [65], and Ter Bogt et al. [66] people use music as a coping method. People engage in this behaviour to maintain emotional control. Listening to music regularly can help people control their effects, regulate their emotions, and boost problem-solving skills [65, 66]. The current study participants reported utilizing music as a coping mechanism to help them manage their stress. This confirms a study by Pouraboli et al. [67], which indicated that participation in a relaxation program offered to a group of parents whose children were suffering from cancer effectively lowered their stress levels. Nyante and Carpenter [68] and Gómez-Zúñiga, et al., [69] claim that family caregivers, especially parents, use their religious or spiritual pursuits as a coping mechanism to get through their trying experiences. This was made possible by having hope in spirituality, having confidence in God, and praying. Similarly, it came to light throughout this research that the participants expressed all hope in their faith, prayed regularly following their religious customs, and had complete faith in God or Allah. The act of faith was consistent with the conviction that only God has the power to heal and that all other attempts are futile in the absence of him. It was observed that prayer, religious practice, and religious expression were all interwoven as sources of inspiration [69, 70]. It also revealed how the road of caregiving was created by strength and faith, which enabled and sustained it. The participants also mentioned that they were able to stay focused and avoid being sidetracked because of their faith in God. This tendency toward optimism seems to be what drives volunteers to keep helping the afflicted and offering whatever support is required. Support for family caregivers of people with terminal illness is a challenge for most professional care providers, due to the nature of the disease progression [71]. Many people would not be able to be cared for at home throughout their final illness if they did not have the support of their family [72]. However, in this study, the findings revealed family caregivers were supported in several ways. Families assisted in providing support to their relatives who served as caregivers. Other family members, religious leaders, and healthcare professionals provided support. However, participants were assisted financially to some extent, though it was revealed that in some cases, it wasn’t enough. Financial compensation for caregiving was deemed to be the most essential kind of support for family caregivers [12]. Support with house chores, cooking, and other errands was provided. This supported a research conducted by [72]which identified that people are more likely to turn to their families for social support in the forms of money, medical care, and other resources than to their communities. In addition, this study is consistent with the findings of a study conducted by Xiuxiang, et al. [73]. in which participants stated that informal support from other family members was available throughout their caregiving role due to filial piety. Every person who holds a religious belief is required to pray regularly [74]. Support from religious groups and representatives was not left out. The research found that they assisted with prayers. Occasionally, representatives from the religious groups were dispatched on behalf of their leaders to help participants and ailing relatives in prayer. This gave the participants additional energy and comfort as they were supported. Words of encouragement were given to cheer them up.

### Implications for practice

The results of this study support the necessity for well-organized palliative care services in underdeveloped nations. The link between palliative services and the needs of caregivers could be established by providing holistic, patient-centered care that focuses not only on the patients but also on the family caregivers. Consequently, the palliative care teams must include multidisciplinary professionals like medical, nursing, social workers, and psychologists who provide emotional and psychosocial support to patients and their caregivers. Coping with the challenges of caring for a terminally ill loved one can be emotionally taxing, and these support services can help caregivers navigate their feelings and concerns. Although health professionals are aware of what palliative care is, its application to the latter still leaves a lot to be desired.

### Strengths and limitations

This study offers a thorough grasp of the difficulties caregivers face and the accessible support systems that can improve their well-being by examining the burden felt by family caregivers of patients with terminal illnesses and proposing helpful coping strategies. Notwithstanding the relevant findings from our study, our inclusion lumped family caregivers of sick relatives diagnosed with terminal illnesses of all conditions at the Palliative Care Unit. It is possible that selecting a specific condition might have shed more light on such conditions than putting them together. Besides, findings from our study can ultimately influence the creation of programs and regulations to provide better support for family caregivers throughout their caregiving journey.

### Electronic supplementary material

Below is the link to the electronic supplementary material.

Supplementary Material 1

## Data Availability

Data and recordings are kept on a drive safely with the corresponding author.
