# Supplementary material for: Challenges and coping strategies when caring for terminally ill persons with cancer: perspectives of family caregivers
Source: BMC Palliat Care. 2024 Jul 17;23:175. doi: 10.1186/s12904-024-01518-z (PMC11253565; doi:10.1186/s12904-024-01518-z)
Supplement: Supplementary file 1 — Supplementary Material 1 [file 12904_2024_1518_MOESM1_ESM.docx]

APPENDIX I**: SEMI- STRUCTURED INTERVIEW GUIDE**

**EXPERIENCES FAMILY INVOLVEMENT IN THE CARE OF PATIENTS WITH TERMINAL ILLNESS; A QUALITATIVE STUDY**

Dear Respondent,

I would like you to answer some questions regarding your role as a care giver for your loved one. You are assured that the answers you give will be strictly confidential and will not be held against you. You are also, free to withdraw or stop answering questions at any time. Thank you.

Respondent ID ………………………………...

Date of interview …………………………………

**SECTION A: SOCIO-DEMOGRAPHIC DATA**

| 1. Background information of patient | 1. Background information of caregiver |
| --- | --- |
| 1. Age: 2. Sex: 3. Religion: 4. Diagnosis: | 1. Sex 2. Age 3. Marital status 4. Employment status 5. Religion 6. Relationship with patient |

**SECTION B: BURDEN OF CAREGIVING**

1. Can you tell me, in what way are you involved in the care of your loved one
2. Describe to me how you feel about being involved in the care of your loved ones
3. Can you tell me how your role as a caregiver has affected you as a person
4. Can you tell me about the challenges you have encountered whilst caring for your loved one
5. What barriers have you faced since you took up the role of caring for your loved one

**SECTION C: COPING STRATEGIES**

1. Can you describe how you have been coping with the barriers and challenges that you have encountered since you started caring for your loved one
2. What other experience can you share with me which we have not mentioned regarding your involvement in the care of loved ones?

We would like to thank you very much for your participation and your opinion.
